# Evaluation of Quality Parameters of Seven Processing Type Potato (*Solanum tuberosum* L.) Cultivars in the Eastern Sub-Himalayan Plains

**DOI:** 10.3390/foods10051138

**Published:** 2021-05-20

**Authors:** Santanu Das, Biplab Mitra, Asok Saha, Somnath Mandal, Prodyut Kumar Paul, Mohamed El-Sharnouby, Mohamed M. Hassan, Sagar Maitra, Akbar Hossain

**Affiliations:** 1 Department of Agronomy, Uttar Banga Krishi Viswavidyalaya, Pundibari, Coochbehar 736165, West Bengal, India; sd.das28@gmail.com (S.D.); bipmitra@yahoo.com (B.M.); asok.ubkv@gmail.com (A.S.); 2 Department of Biochemistry, Uttar Banga Krishi Viswavidyalaya, Pundibari, Coochbehar 736165, West Bengal, India; smandal8183@gmail.com; 3 Department of Pomology and Post-Harvest Technology, Uttar Banga Krishi Viswavidyalaya, Pundibari, Coochbehar 736165, West Bengal, India; prodyut24@yahoo.com; 4 Department of Biotechnology, College of Science, Taif University, P.O. Box 11099, Taif 21944, Saudi Arabia; m.sharnouby@tu.edu.sa; 5 Department of Biology, College of Science, Taif University, P.O. Box 11099, Taif 21944, Saudi Arabia; 6 Department of Agronomy, Centurion University of Technology and Management, Paralakhemundi 761211, Odisha, India; sagar.maitra@cutm.ac.in; 7 Department of Agronomy, Bangladesh Wheat and Maize Research Institute, Dinajpur 5200, Bangladesh

**Keywords:** dry matter, specific gravity, chips colour score, starch, potato

## Abstract

The eastern sub-Himalayan plain of India is a popular potato growing belt in which vast scope exists to introduce processing grade cultivars. The selection and introduction of a better quality processing grade cultivar in this region may pave the way for the processing industries. Keeping these in the backdrop, this study was conducted at Instructional Farm of Uttar Banga Krishi Viswavidyalaya (UBKV), Pundibari, Cooch Behar, West Bengal, India under eastern sub-Himalayan plains during winter seasons of 2016–17 and 2017–18 in which seven processing type potato cultivars (Kufri Chipsona-1, Kufri Chipsona-3, Kufri Chipsona-4, Kufri Frysona, Kufri Himsona, Kufri Surya and Kufri Chandramukhi) were evaluated in terms of different quality parameters pre-requisite for chips processing viz., dry matter content, specific gravity, starch content, chips colour score, crispiness and hardness of chips through randomised complete block design (RCBD). The study revealed wide variation in all quality parameters amongst the cultivars. Cultivar ‘Kufri Frysona’ showed the highest specific gravity (1.121) as well as dry matter content (23.35%) followed by ‘Kufri Chipsona-3’. The cultivar ‘Kufri Frysona’ showed the highest starch content (28.52%) too. Chips prepared from ‘Kufri Chipsona-1’ were recorded to be crispier with a relatively lower value of deformation before the first break and less hardness value. All processing type potato cultivar reflected the chips colour score <3 (evaluated, based on 1–10 scale, 10 being the darkest and least desirable) though ‘Kufri Frysona’ had the lowest chips colour score (1.50) signifying its superiority for the region. ‘Kufri Frysona’ cultivation could be recommended in this agro-climatic region particularly for chips manufacturing potato industries.

## 1. Introduction

Potato (*Solanum tuberosum* L.) is a world food crop having a significant contribution towards food and nutritional security, especially in the developing world. It is considered as a balanced food due to the presence of high-quality proteins, vitamins and minerals, trace elements with lesser energy [1]. It contains some important essential amino acids like leucine, tryptophan and isoleucine. The demand for processed potato products like chips, french fries, lachcha, flakes etc. is ever increasing due to rapid urbanisation, an inclination of the young generation towards fast foods, higher income, increasing number of working women with their preference towards ready-cooked food and tourist demand. Even though India is the third-largest producer in the world, mostly table purpose potato is grown in the country with a little share of processing type potatoes. The table purpose cultivars had low dry matter (17–19%) and high reducing sugar (>250 mg 100 g^−1^ fresh tuber weight) content which in turn reflected poor chips colour score and crispiness. These table purpose cultivars were not preferred by the industries though sometimes they used the table purpose potato for making crisps and french fries due to lack of availability of suitable cultivars. However, the market for processed potato products is getting momentum in the last few years [2] and consequently, the demand for processing type potato cultivars by the processing industries has enhanced a lot. It has been estimated that the demand for processing type potato with desirable traits would rise up to 25 million tonnes by 2050 [3].

To augment the supply of processing-grade potatoes for the industry, ICAR-Central Potato Research Institute (CPRI), India is continuously engaged in developing improved processing grade cultivars and in 2005, ‘Kufri Chipsona-3’ was released, which showed higher tuber yield with a better processing quality over ‘Kufri Chipsona-1’ and Kufri Chipsona-2, the earlier released processing cultivars [4,5]. Based on the trials conducted over different parts of India, it was seen that these processing cultivars are higher yielder (>30 t ha^−1^), high in dry matter content (21–24%), low in reducing sugar content (<0.1%), low in glycol-alkaloids and phenols, lesser undesirable colour (<5%) and total chips defects (<15%) [6]. It is well known that dry matter content, specific gravity, reducing sugar content (glucose and fructose) as well as chips colour score are the important quality traits for potato. Being the decisive factor for product recovery and oil content in chips, tuber dry matter is the most important characteristic. Reducing sugars also influence processing because of the ‘Maillard reaction’ [7] between amino acids and reducing sugars at the time of frying. Tuber maturity, growing condition, water and nutrient uptake were the other determining factors of tuber dry matter and reducing sugars in potato [8].

Considering the better scope of marketing of chips potato in north-eastern states of India as well as in neighbouring countries, Bhutan and Nepal in particular, eastern sub-Himalayan plains of West Bengal, India may serve as a potential belt where potato is grown over sixty thousand hectares of land and an automatic choice of farmers during winter months [9]. In spite of high production, the processing type cultivars were not evaluated till date, in this region. Moreover, the region is accompanied with high rainfall, high humidity, prolonged winter season and acidic soil pH. We speculate chips related biochemical parameters may get influenced by the genotypes, growing conditions, season, soil types, amount of rainfall received, other prevailing environmental and storage conditions and their inter-relationship. The performance and evaluation of processing type potato cultivars with chips making quality will be an interesting study for this region which is basically a ‘non-conventional potato processing belt’. Keeping these in the background, the present work has been designed to identify processing type potato cultivars with acceptable qualities and chemical composition pre-requisite for chips processing with respect to dry matter content, specific gravity, starch content, chips colour score, crispiness and hardness of chips; suitable for this agro-climatic region.

## 2. Materials and Methods

### 2.1. Site Description

The present investigation was carried out at the Institutional Farm of Uttar Banga Krishi Viswavidyalaya, Pundibari, Cooch Behar, West Bengal, India during the winter season of two consecutive years viz., 2016–17 and 2017–18. Geographically the experimental area was situated at eastern sub-Himalayan plains having 28°58′86″ N latitude, 81°66′73″ E longitudes and an altitude of 43 m above the mean sea level. This area is characterised by high rainfall, high humidity and prolonged winter. The long-term data suggested an overall temperature range of 7.1–8.0 °C (minimum) to 24.8–32.2 °C (maximum) for the region. During the experimental period, the maximum and minimum temperature fluctuated between 24–30 °C and 8–15 °C in 2016–2017; while it was ranging between 23–30 °C and 8–16 °C in 2017–2018, respectively. The total rainfall during the crop growing period (November to March) was 48.00 mm (2 rainy days) and 4.71 mm (2 rainy days) in 2016–2017 and 2017–2018, respectively. In general, the cool climatic condition during December-January months was favourable for tuber formation and development. The soils at the research sites were sandy loam in texture with acidic pH (5.77) having low mineralisable nitrogen (132.78 kg ha^−1^), medium available phosphorus (27.96 kg ha^−1^) and low available potassium (142.86 kg ha^−1^). The available nitrogen was estimated through the hot alkaline permanganate method [10]. The available phosphorus (P) was extracted with 0.5 M NaHCO_3_ (pH 8.5) and estimated through a UV-VIS spectrophotometer [11]. Available fraction of potassium (K) was extracted with neutral normal ammonium acetate (pH 7.0; 1:10 *w*/*v*) solution and estimated through a Flame photometer [12].

### 2.2. Experimental Treatments and Design

Seven processing type potato cultivars (Kufri Chipsona-1, Kufri Chipsona-3, Kufri Chipsona-4, Kufri Frysona, Kufri Himsona, Kufri Surya and Kufri Chandramukhi) were taken in the experiment. All the selected cultivars are having desirable processing characteristics like easy to cook, waxy texture, mild flavour, free from after-cooking discolouration with high dry matter and low reducing sugar content. The experiment was laid out in a randomised complete block design (RCBD); each cultivar replicated thrice with a net plot size of 3.6 m × 4.5 m. The land was thoroughly prepared and well-sported tubers of each cultivar were planted on ridges spaced at 50 cm, with a plant to plant distance of 15 cm. Spacing between plots and replication were kept at 1 and 1.5 m, respectively.

All the varieties were fertilised with 125 kg N ha^−1^, 100 kg P_2_O_5_ ha^−1^ and 125 kg K_2_O ha^−1^ in the form of urea, single super phosphate and muriate of potash, respectively. Out of these nutrient doses, 1/2 of nitrogen and a full dose of P_2_O_5_ and 2/3 of K_2_O were applied as basal on the date of planting of tubers. The rest half of nitrogen was applied in two equal splits, first top dressing at 21 days after planting (DAP) and the remaining portion of N along with the remaining 1/3 K_2_O was applied as the second top dressing during 42 DAP. During the entire crop production cycle, cultural practices including hoeing, hilling and irrigation were carried out uniformly on all plots. Ten days before harvesting the haulm was removed using sickles and the tubers were harvested by splitting the ridges with country plough.

### 2.3. Data Collection

The observations were recorded on seven different biochemical parameters viz., starch, crispiness and hardness of chips, tuber dry matter content, specific gravity, reducing sugars and chip colour score from five randomly selected plants and the average was subjected to statistical analysis of variance.

#### 2.3.1. Specific Gravity

The specific gravity of potato tubers was determined by the procedure given by Westermann et al. [8]. Specific gravity was calculated using the formula.

$$\mathrm{Specific}\;\mathrm{gravity}\;=\frac{\mathrm{Weight}\;\mathrm{of}\;\mathrm{tuber}\;\mathrm{in}\;\mathrm{air}}{\mathrm{Weight}\;\mathrm{of}\;\mathrm{tuber}\;\mathrm{in}\;\mathrm{air}\;-\;\mathrm{Weight}\;\mathrm{of}\;\mathrm{tuber}\;\mathrm{in}\;\mathrm{water}}$$

#### 2.3.2. Dry Matter Content in Tubers

Tuber dry matter content was determined by randomly taking ten tubers from the harvested plot. Two sub-samples of 200 g each were taken after washing, chopping and mixing and pre-dried at a temperature of 60 °C for 15 h and further dried for 3 h at 105 °C with the help of a drying oven. The ratio between dry and fresh mass was calculated as the dry matter content and was expressed in percentage.

#### 2.3.3. Determination of Starch Content

To determine the starch content, a fresh sample weighing 0.1 to 0.5 g was homogenised in hot 80% ethanol followed by centrifugation at 10,000 rpm for 20 min. After that 5 mL of water and 6.5 mL of perchloric acid was added to the residue and refrigerated at 0 °C for 20 min. The residue was centrifuged again and the supernatant was saved for analysis. The final volume was made up to 100 mL and dilution was made in the ratio 1:5 using distilled water. 4 mL of anthrone reagent was added to each test tube and heated for about 8 min in a boiling water bath. The content was rapidly cooled and the intensity of green to dark green was read in UV visible spectrophotometer (Thermo Spectronic, USA, model Genesys 10) at 630 nm. A series of glucose working standards solution (20–100 µg mL^−1^) were used for calculating the starch content of the fresh potato sample [13].

#### 2.3.4. Determination of Reducing Sugar Content

For reducing sugar extraction, 100 mg of fresh sample weighed in a centrifuge tube and sugars were extracted with the addition of hot 80% ethanol twice and centrifuged at 10,000 rpm for 20 min, the supernatant was collected and evaporated in a water bath, then 10 mL of water was added. 1 mL of aliquot is taken from each test tube and equalised with 3 mL of water followed by the addition of 3 mL of DNS reagent and kept in a boiling water bath for 5 min. After the development of the colour, 1 mL of 40% Rochelle salt solution was added and mixed thoroughly. After cooling, absorbance was measured in UV visible spectrophotometer (Thermo Spectronic, USA, model Genesys 10) at 510 nm. The standard curve was prepared with glucose solutions (20–100 μg) [13].

#### 2.3.5. Blanching Treatment

Blanching of potato is recommended practice for desirable frying at the industrial level. It kills the potential food pathogens, deactivates the enzymes causing discolouration, ensures a longer storage period, reduce the moisture content of fries and thereby making them crispier, and improves the taste and texture of the fried potato. Chips were prepared 15 days after the harvest of potato tuber. During these 15 days, the potato was stored in an ambient environment (well-aerated, cool, dark, humid environment with an average 18–21 °C temperature to prevent the conversion of starch to sugars, greening and sprouting). Randomly selected 10 healthy and undamaged potato tubers from each replication were washed and peeled before slicing. The average thickness of slices was kept at 1.5 to 1.8 mm with a hand-operated slicer. The slices were blanched for 1 min at 60 °C and cooled in water at room temperature. The slices were wrapped in a paper towel to remove surface water.

#### 2.3.6. Frying Processes

For frying potato slices, the procedures described by Ezekiel et al. [14] were employed with slight modifications. Initially, the frying oil (refined sunflower oil) was heated for about 15–20 min until the required temperature of 180 °C is reached which was monitored by a thermometer. The ratio of the frying oil and potato slices were maintained at 20:1 uniformly throughout the frying process. 500 g slices of previously blanched potato were fried using a fryer (Beckers, 24,047) until bubbling ceases (3–4 min), after that the fried strips were removed from the oil, drained for 1 min and air-dried at 20–22 °C for 5 min on a paper towel. The experiment was repeated three times for each cultivar.

#### 2.3.7. Packaging and Storage of Chips

The chips packed in a HDPE pouch of 8 gauze thickness. Around 25 g of chips were packed in a pouch with 635.5 sq cm surface areas. The chips were stored in the ambient environment and the temperature and relative humidity of the environment was recorded. It was observed that during the period of storage of chips from 12th March (11th Standard Meteorological Weeks) to 3rd June (21st Standard Meteorological Weeks), the mean maximum and minimum temperature varied between 26.7–33.2 °C and 15.2–24.1 °C during 2017 and 29.3–32.2 °C and 16.4–22.9 °C during 2018, respectively. During this period, the relative humidity ranged between 67–93% (higher values during May-June due to the onset of monsoon showers). The crispiness and hardness of the chips were measured at 15 days after frying (DAF) of chips.

#### 2.3.8. Chips Colour Measurement

Fried chips were evaluated for chips colour score on a scale of 1–10 [14], subjectively with the help of colour cards where 1 denotes a highly acceptable colour and 10 denotes a dark brown unacceptable colour, while chips with a colour range of up to 4.0 were considered acceptable.

#### 2.3.9. Measurement of Crispiness and Hardness

Crispiness and hardness measurements of the chips were performed using Texture Analyzer (TA-XT2i, Stable Micro Systems Ltd., Surrey, UK) [15]. A crisp fracture rig (HDP/CFS) and a cylindrical probe (P/2N) having a 2 mm diameter were used for the measurement. Experiments were carried out by placing the fried chips samples on the rig and by driving the probe perpendicularly attached to the crosshead with a speed of 1 mm s^−1^. Texture measurements were performed approximately 2 h after the potato chips were removed from the frying oil. Three potato chips were analysed for each replication and the average data were recorded. From the force-deformation curve, the following parameters were obtained; maximum force value (force at the first major drop, Fmax, N) and deformation value (deformation-induced at maximum force, mm).

The crispiness of the chips was measured as distance (mm) travelled by probe before the first break which was indicated by the sharp drop in force. Chips were considered to be crispier if the value of distance was smaller.

Hardness was measured as the pressure exerted by the chips to the probe at the first break. It was calculated by dividing the maximum force measured at first break by the area of the probe in contact with the chips.

### 2.4. Statistical Analysis

The analysis of all data was performed with the help of the analysis of variance (ANOVA) technique for RCBD. The least significant difference test was used to compare the effects of treatments at a 5% level of significance. The mean values were adjudged by the Duncan Multiple Range Test (DMRT) using SPSS version 20.0.3. In addition, the relationships among all quality parameters (dry matter, starch, reducing sugar, chips colour, hardness and crispiness) were assessed using bivariate correlation analysis (Pearson correlation coefficients and a two-tailed test of significance).

## 3. Results and Discussion

### 3.1. Dry Matter and Specific Gravity of Different Processing Cultivars

Dry matter contents and specific gravity were the most important quality parameters for potato processing influencing crispiness, hardness, flavour, colour and processing efficiency [16,17]. It was observed that the processing type potato cultivars did not vary significantly in terms of specific gravity which ranged between 1.110 to 1.121 (Table 1). Amongst the cultivars, ‘Kufri Frysona’ showed the highest specific gravity (1.121) followed by ‘Kufri Chipsona-3’ (1.117). This difference in specific gravity might be related to genetic variations and variation of dry matter content among different cultivars [18]. ‘Kufri Frysona’ showed the highest dry matter content (23.35%) than other cultivars. This was in the line of conformity with Kaur and Aggarwal [18], who were in the opinion that the genotype had a direct influence on the dry matter content.

### 3.2. Crispiness and Hardness of Chips Prepared from Different Processing Cultivar

The cultivars differed significantly (*p* < 0.05) in terms of crispiness and hardness. Chips prepared from ‘Kufri Chipsona-1’ reflected more crispiness with a relatively lower value of deformation before the first break and less hardness value [0.734 mm and 11.39 kg cm^−2^ at 15 days after frying (DAF), respectively]. However, ‘Kufri Chipsona-3’ (0.763 mm) and ‘Kufri Chipsona-4’ (0.791 mm) were observed to be less crispy with a relatively higher value of deformation before the first break in comparison to ‘Kufri Chipsona-1’. In general, the crispiness and hardness were attributed to the influences of dry matter content [19]. Generally, the chips prepared from tubers having high dry matter content had a weak structure compared to chips prepared from tubers with the lower dry matter. The crispiness and hardness of potato chips were found to be directly related to the starch content of tubers also. The lower value of crispiness and hardness achieved with the processing type potato cultivars were found to be rich in dry matter (above 20% and varies between 20.5 to 23.35%) [20]. This study is in the line of conformity with the findings of Moyano et al. [21] and Lisinska and Leszynski [22]. Jaswal [23,24] investigated how crispiness varied with specific gravity and polysaccharide content, the major determining factors for making the chips crispy. As the polysaccharides differed in their molecular mass, the chips prepared from the potatoes having higher specific gravity did not subject to polysaccharide degradation and thus showed better texture. The non-starch polysaccharide having higher molecular mass was obtained from the tubers having higher specific gravity than the potatoes of lower specific gravity [20].

### 3.3. Starch Content of Different Processing Cultivars

There was a wide variation in starch content (23.67 to 28.52%) amongst the cultivars studied (Table 1). The cultivar ‘Kufri Frysona’ showed the highest starch content (28.52%) followed by ‘Kufri Himsona’ (26.80%) and ‘Kufri Chipsona-3’ (26.41%). The starch contents in table purpose potato tubers mostly ranged between 11 to 17%, while the value was much higher (18–22%) in high starch potatoes [25,26]. In this study, the higher starch content may be attributed due to the genotype X environment interaction effect manifested by the cultivars. It was reported that the total starch concentration was affected by cultivar or growing location including environmental conditions and cultural practices during the growing season [27,28]. There was a strong relationship between the total starch and dry matter contents. Starch contents were also related to specific gravity, which could also influence potato processing quality. Actually, the starch degraded rapidly into reducing sugar under low-temperature storage condition called low-temperature sweetening [29]. The physiological ageing of the tubers by the beginning of the sprouting process could also increase the reducing sugar content [30].

### 3.4. Reducing Sugar and Chips Colour Score of Different Processing Cultivar

**Figure 1:**
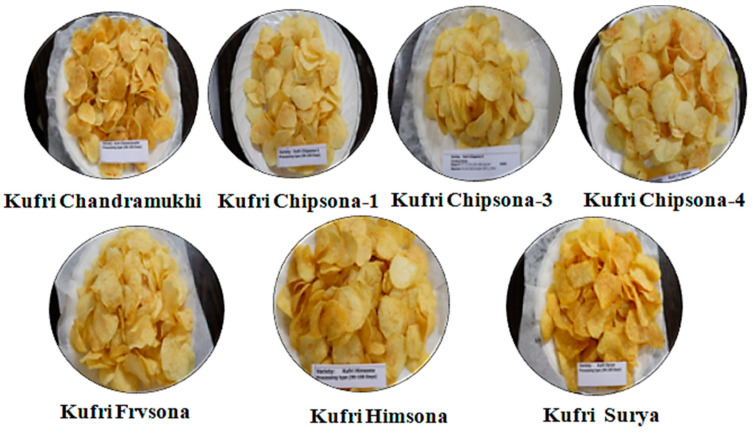
Chips colour of various processing type cultivar 15 days after frying.

The lowest reducing sugar content and chips colour score were observed in ‘Kufri Frysona’ (82.40 mg 100 g^−1^ fresh weight and 1.50), closely followed by ‘Kufri Chipsona-3’ and ‘Kufri Chipsona-4’ (Table 1). Sugar content in potato tuber is a heritable character and may be affected by cultivar, maturity, season and production site and storage conditions. For processing purpose, low reducing (<0.1%) and total (<2.0% on fresh weight basis) sugars are desirable to avoid dark colour and bitter taste in processed potato products. It has been reported that the high or low dose of reducing sugars gets influenced by variety, cultural and environmental conditions [31]. The higher reducing sugar content in this experiment could be attributed to the receipt of heavy rainfall (37.40 mm) during later stages of crop growth. The variation of reducing sugars of the same cultivars in different growing conditions was reported by Nelson et al. [32]. The results of chips colour, when the tuber was harvested at 100 DAP, revealed that the processing type varieties gave light and acceptable chips colour score (1–3) (Figure 1) and these varieties had higher dry matter content (>20%) with low reducing sugars (<100 mg 100 g^−1^ fresh weight) in tubers. It was also observed from the results that the processing type cultivars had a comparatively higher specific gravity (>1.1) and starch content (>23%) leading to the acceptable and finest colour of chips.

A positive correlation of dry matter content (Table 2) with specific gravity (r = 0.925 **) and starch content (r = 0.841 *) was observed in this study. Table 2 also indicated a negative correlation of specific gravity with reducing sugars (r = −0.787 *). Again, the reducing sugar content had a significant and positive correlation with chips colour (r = 0.828 *). The results corroborated with the findings of Ezekiel et al. [14] and Kumar et al. [33]. A significant relationship between dry matter and specific gravity had also been reported previously [34]. Manivel et al. [31] reviewed that potato should have >20% dry matter content for chips and processing or industrial purposes. The acceptable range of dry matter content of potato for chips had been reported to be 17.19% to 22.99% in the Netherlands [35]. However, in India, ICAR-CPRI accepted that the dry matter content should be more than 20%. Similar findings were reported by Kumar et al. [36] in India, where they reported a positive correlation between starch, specific gravity and dry matter content based upon a regression equation.

## 4. Conclusions

From this two-years trial, it can be concluded that the cultivar ‘Kufri Frysona’ showed the highest dry matter (23.35%) and starch content (28.52%) with the lowest chips colour score (1.50) (on a scale of 1–10) reflecting its superiority amongst the cultivars. Considering its superior quality parameters, this cultivar could be recommended for chips manufacturing potato industries under the sub-Himalayan plains of India.

## Figures and Tables

**Table 1:** The quality parameters of different processing type potato cultivars.

Cultivars	Specific Gravity	Tuber Dry Matter Content (%)	Crispiness of Chips (mm) at 15 DAF	Hardness of Chips(kg cm^−2^) at 15 DAF	Starch (%)	Reducing Sugar (mg 100 g^−1^ Fresh wt.)	Chips Colour Score
Kufri Chipsona-1	1.110	20.63 ab	0.734 a	11.39	25.15 a	94.20 b	2.50 a
Kufri Chipsona-3	1.117	22.07 bc	0.763 ab	13.83	26.41 abc	87.57 ab	2.17 a
Kufri Chipsona-4	1.112	20.25 a	0.791 b	13.06	23.67 a	92.00 b	2.00 a
Kufri Frysona	1.121	23.35 c	0.815 bc	13.69	28.52 c	82.40 a	1.50 a
Kufri Himsona	1.116	22.01 bc	0.948 e	12.77	26.80 bc	89.26 ab	2.33 a
Kufri Surya	1.112	20.50 a	0.875 d	13.89	24.43 ab	109.11 c	2.83 ab
Kufri Chandramukhi	1.108	20.60 ab	0.859 cd	13.93	24.95 ab	105.68 c	4.00 b
LSD (*p* ≤ 0.05)	NS	**	**	NS	**	**	**

NS—Non-Significant; DAF—Days after frying (of chips); Numbers followed by various lower-case letters within a column are significantly different from each other at *p* ≤ 0.05 and are otherwise statistically on par; ** significant at *p* ≤ 0.01 level.

**Table 2:** Correlation studies for various quality parameters of different processing type cultivars.

Characters	Tuber Dry Matter	Starch	Reducing Sugar	Chips Colour	Crispiness of Chips	Hardness of Chips
Specific gravity	0.925 **	0.841 *	−0.787 *	−0.799 *	−0.544	−0.294
Tuber Dry matter		0.979 **	−0.751	−0.587	−0.442	−0.313
Starch			−0.693	−0.483	−0.400	−0.363
Reducing sugar				0.828 *	0.733	0.718
Chips colour					0.796 *	0.570
Crispiness of chips						0.579

** Correlation is significant at the 0.01 level (2-tailed); * correlation is significant at the 0.05 level (2-tailed).

## Data Availability

Data may be available after request.
